# Detection of Virulence Genes and Antimicrobial Susceptibility Profiles of *Staphylococcus aureus* Isolates From Bovine Mastitis in Chagni, Northwestern Ethiopia

**DOI:** 10.1155/vmi/6473601

**Published:** 2025-02-17

**Authors:** Ahmed Wodaje, Mequanint Addisu Belete, Ashenafi Syoum Menkir, Zemenu Birhan Zegeye, Fanuel Bizuayehu Yihunie

**Affiliations:** ^1^Chagni Town Administration and Development Office, Municipal Abattoir, Chagni Town, Awi Zone, Ethiopia; ^2^Department of Veterinary Laboratory Technology, College of Agriculture and Natural Resource, Debre Markos University, Debre Markos, Ethiopia; ^3^Department of Veterinary Medicine, College of Veterinary Medicine and Animal Science, Samara University, Semera, Ethiopia; ^4^College of Agriculture and Environmental Science, Bahir Dar University, Bahir Dar, Ethiopia

**Keywords:** antibiotics, bovine mastitis, Chagni, *Staphylococcus aureus isolation*, Virulent gene detection

## Abstract

Mastitis is an inflammation of the mammary gland tissue that is generally associated with an infection. *Staphylococcus aureus* is a primary pathogen responsible for bovine mastitis worldwide. Nonetheless, there is limited information on virulence factors and resistance profile of *Staphylococcus aureus* associated with bovine mastitis in northwestern Ethiopia. This study aimed to determine the frequency of virulence genes and antibiotic susceptibility profile of *Staphylococcus aureus* in dairy cows with mastitis. A cross-sectional study with a simple random sampling method was conducted from October 2022 to June 2023 in Chagni town, Amhara region from a ranch and 20 smallholder farms. *Staphylococcus aureus* was isolated and identified using standard bacteriological and molecular methods, followed by antibiotic sensitivity testing of the isolates. Descriptive statistics was used to summarize the study results. Of 140 milk samples tested, 64 (45.7%) were positive for *Staphylococcus aureus*. Enterotoxins (*seb* = 13 [20.3%], *sec* = 11 [17.2%], *seh* = 9 [14.1%], and *see* = 6 [9.4%]), Panton–Valentine leukocidin (*pvl* = 11 [17.2%]), toxic shock syndrome toxin (*tst* = 7 [10.9%]), and alpha-hemolysin (*hlb* = 7 [10.9%]) were the prominent virulence genes. The isolates exhibited high antimicrobial sensitivity to sulfamethoxazole (87.5%) and gentamycin (79.7%), followed by tetracycline (75%), erythromycin (72%), and azithromycin (71.8%). However, they were highly resistant to cefoxitin (65.6%), followed by erythromycin, tetracycline, and ciprofloxacin (25%). Multidrug resistance was also observed in 23 isolates, which showed resistance to at least one agent in three or more antimicrobial categories. Our research identified a significant presence of virulent genes and antibiotic-resistant *Staphylococcus aureus* responsible for mastitis, underscoring the critical necessity for enhanced specific mastitis control strategies against *S. aureus* in the study setting.

## 1. Introduction

The dairy industry in Ethiopia has the potential to significantly reduce poverty and malnutrition for many rural residents and a considerable number of urban and peri-urban dwellers [1]. However, the country's milk and dairy needs are still unmet due to inadequate production due to disease and a backward production system. Among the diseases, mastitis is one of the main contributors to the decrease in milk production [2]. The disease is commonly caused by bacteria such as *Staphylococcus aureus*, *Streptococcus dysgalactiae*, *Streptococcus agalactiae*, *Streptococcus uberis*, and *Escherichia coli* [3].

*Staphylococcus* species are common causes of bovine mastitis in dairy herds. Pathogenic strains, usually coagulase-positive, have been found to cause disease in a wide range of hosts, including humans, worldwide [4]. The pathogenic strains of *S. aureus* are particularly prevalent pathogens among staphylococci species isolated from intramammary infections in dairy cows [5]. The prevalence of *S. aureus* in bovine mastitis can vary depending on several factors, including geographical location, management practices, and herd health status. In some regions, it is the primary cause of mastitis, while its occurrence is minimal in others [6]. While, *S*. *aureus* infections can be treated with various antibiotics, the extensive use of antibiotics for treatment or as growth promoters in livestock has led to the spread of resistance in bacterial pathogens, impacting both human and animal health [7]. Oxacillin, methicillin, and other beta-lactam antibiotics are no longer effective against *S*. *aureus* [8–10]. In a study conducted in Ethiopia, pathogens causing various disease conditions showed resistance ranging from 30% to 85% to antimicrobial products listed in the Ethiopian Standard Treatment Guidelines (ESTG). In addition, resistance to specific drugs such as penicillin, ampicillin, or amoxicillin reached up to 100% in some reports [11].

The detection and antibiotic susceptibility profiles of *S*. *aureus* causing bovine mastitis have not yet been investigated in the current study area. In Ethiopia, there is a significant problem with the misuse of veterinary drugs. Many prescriptions are performed without proper diagnostic support, leading to inappropriate antimicrobial use. In addition, most prescriptions are made by animal health assistants rather than veterinarians, which can result in improper prescription [12]. Research on the presence of *S*. *aureus* in cows with mastitis, along with antimicrobial susceptibility testing, is essential for effective management and selecting suitable treatment options in the study area. Therefore, this study aimed to determine the frequency of virulence genes and the antibiotic sensitivity profile of *S*. *aureus* isolates from dairy cows with mastitis.

## 2. Materials and Methods

### 2.1. Description of the Study Area and Farms

**Figure 1:**
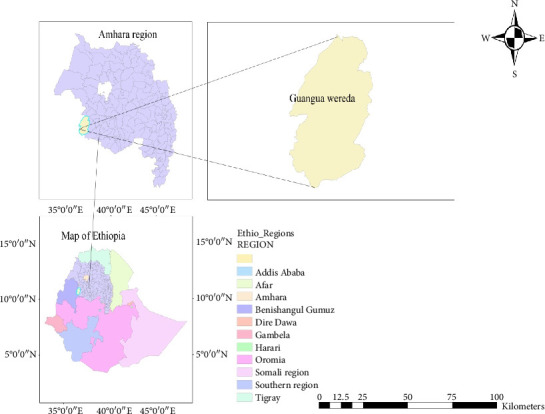
Map showing the location of the study area where sampling was conducted. Maps were generated using ArcGIS software Version 10.8.

The study was conducted in Chagni, Guanga district of Awi Zone, Amhara Regional State, Ethiopia (Figure 1). The areas were chosen due to the high potential of farm animals in this district, with ample grazing land and a favorable climate. The farms selected for the study were Chagni cattle breeding and improvement ranch and smallholder dairy farms. The criteria for the selection of farms were based on the available dairy cattle, the willingness of farm owners to participate, access to the farm location, and the cooperation of government officials in providing necessary permits and support.

Chagni cattle breeding and improvement ranch was founded in 1986 E. C., focusing on conserving and improving the Fogera cattle breed. It is currently involved in raising livestock on 1367 hectares of land with a large scale of operation. The ranch implements sustainable grazing practices to ensure the health and productivity of its animals. Smallholder dairy farms often operate with limited resources under poor hygiene, management, and disease control practices. The housing in the ranch and smallholder dairy farms is mainly earthen and concrete floors with shade and exercise barns around the house.

### 2.2. Study Design, Sample Size, and Sampling Methods

A cross-sectional study was conducted between October 2022 and June 2023. The sample size was determined following the method described by Thrusfield [13]. Therefore, the sample size was determined at a 95% confidence interval with 5% precision based on previous studies in the study area [14] and with an expected prevalence of 28.2%.

(1) $$\begin{array}{c} n=\frac{1.96^{2}\left(P_{e\text{xp}}\right)\left(1\;–\;P_{e\text{xp}}\right)}{d^{2}},\\ n=\frac{1.96^{2}\left(0.282\right)\left(1\;–\;0.282\right)}{0.05^{2}},\\ n=318, \end{array}$$

where *n* is the sample size, 1.96 is the value of “*Z*” at a 95% confidence interval, *P*_exp_ is the expected prevalence, and *d* is the desired absolute precision.

For this study, 318 lactating cows were randomly selected, including 212 from Changi cattle breeding and improvement ranch and 106 from smallholder dairy farms. The study animals were included based on the proportion of cattle in each production system. Both lactating cows that were experiencing clinical symptoms of mastitis, such as swelling, heat, redness, hardness of the udder, and reduction of milk yield, and cows with no observable clinical signs were included in the study. Contrarily, lactating cows who had received systematic or inflammatory antimicrobials and topical treatment in the last 2 weeks were excluded.

### 2.3. Sample Collection and Bacteriological Analyses

Milk samples were collected from all quarters of the cow's udder that exhibited clinical or subclinical mastitis. 140 milk samples were collected and subjected to bacterial isolation from 26 clinical and 114 subclinical mastitic cows at Chagni cattle breeding and improvement ranch and smallholder dairy farms. Approximately 10 mL of milk was collected in sterile tubes. The samples were then transported to the Institute of Biotechnology, Bahir Dar University, for bacteriological analysis. A loopful of milk samples was plated onto blood agar (Oxoid, Hampshire, United Kingdom) with 5% sheep blood and further subcultured on mannitol salt agar (MSA) (HiMedia, India). The agar plates were incubated at 37°C for 48 h and examined every 24 h to determine the optimum growth of bacterial colonies. Colonies with distinct hemolytic properties (clear beta-hemolysis) and morphology (small yellow colonies on MSA) were selected as presumptive *S*. *aureus* and purified using nutrient agar plates.

Biochemical assays (Gram staining, catalase, and tube coagulase tests) were performed to identify catalase- and coagulase-positive *Staphylococcus* species from catalase-negative *Staphylococcus* species, coagulase-negative *Staphylococcus* species, and other *Streptococcus* species [15]. In addition, an oxidation–fermentation test was used to differentiate *Staphylococcus* from *Micrococcus* species. Each presumptive *S. aureus* isolate containing glycerol was stored at −20°C in Tryptone Soya Broth (TSB) (HiMedia, India) for further molecular analysis.

### 2.4. DNA Extraction

The genomic DNA of each presumptive *S. aureus* isolate was extracted by commercially available Bio Basic Genomic DNA Extraction Kit (Bio Basic Group, Canada). In brief, 1.5 mL of overnight grown culture in TSB was pelleted at 10,000 rpm for 5 min. The pellet was washed twice with 1x phosphate buffer saline (PBS) at 8000 rpm for 3 min. Next, the pellets were collected and resuspended in 200 μL of cold TE (10 mM Tris-Hcl, 1 mM EDTA, pH 8.0), 400 μL of digestion solution, and 3 μL of Proteinase K solution (2 mg/150 μL) and incubated at 55°C for 5 min following manufacturer's instructions. The quality and quantity of the DNA extracted were assessed using 1% agarose gel (Bio Basic, Canada) and a NanoDrop 2000 Spectrophotometer (Thermo Scientific TM, USA), and DNA was stored at −20°C for subsequent analyses.

### 2.5. Molecular Confirmation of *S. aureus* and Detection of Virulence Genes

Confirmation of *S. aureus* was performed by PCR amplification of the conserved thermostable nuclease gene (nuc) according to primer sets and PCR conditions described previously [16]. All nuc*-*positive isolates were further screened for the detection of the following genes encoding enterotoxins (sea, seb, sec, sed, see, and seh); Panton–Valentine leukocidin (*pvl*); toxic *shock* syndrome toxin (tsst-1) (*tst*); and alpha-hemolysin (*hlb*). Amplifications were carried out in the LongGene A300 Fast Gradient Thermal Cycler (LongGene Scientific Instruments, China). The 25 μL PCR mixture volume contained 12.5 μL of master mix (2x PCR Master Mix, Thermo Scientific, United States of America), 0.5 μL of each forward and reverse primer (Table 1), 2.5  μL of template DNA, and 9 μL nuclease-free water. The thermal conditions used for gene amplification were programmed according to the previous protocol [16, 21–25]. The PCR products were separated by electrophoresis in 1.5% agarose (w/v) in 1x TAE buffer (40 mM Tris-HCl, 20 mM acetate, 0.5 mM EDTA, and pH 8.3). The molecular marker 100 base pair DNA ladder (HiMedia, India) was used in all electrophoresis. The gel result was stained with ethidium bromide and photographed under a gel documentation machine (Bio-Rad, Germany). The target gene, primer sequences, expected product size, and amplification conditions are shown in Table 1.

### 2.6. Antimicrobial Susceptibility Testing

All 64 isolates of *S*. *aureus* from mastitic milk were tested for antimicrobial susceptibility to eight selected antimicrobials. The sensitivity of isolates to different antibiotic agents was determined by the disk diffusion method using commercial disks and interpreted based on standards [26]. Seven antibiotic classes comprising lincosamides (clindamycin [2 μg]), macrolides (erythromycin [15 μg] and azithromycin [25 μg]), aminoglycosides (gentamicin [10 μg]), sulfonamides (sulfamethoxazole [25 μg]), tetracycline (tetracycline [30 μg]), fluoroquinolones (ciprofloxacin [5 μg]), and cephalosporin (cefoxitin [30 μg]) were used for the sensitivity test. The recommendation for treatment of the isolated bacteria, local epidemiological data on infections and drug usage, antibiotics with a broad spectrum of activity, the availability and cost of antibiotics in the local clinics, and antibiotic resistance trends guide the selection of antibiotics.

Pure colonies of *S. aureus* cultures were inoculated in saline water (direct colony suspension) to get turbidity equal to 0.5 on the McFarland scale (108 CFU/mL). Then, a sterile cotton swab was used to inoculate the bacteria on the Mueller–Type equation here.Hinton agar medium. Antibiotic discs were applied to the medium using sterile forceps and pressed gently to ensure complete contact with the agar surface. Finally, the plates were incubated at 37°C and examined after 24 h for the presence or absence of inhibition zones [12]. Multidrug resistance was determined based on the isolate's resistance profiles to at least one agent in three or more antimicrobial categories. The isolates were classified as sensitive, intermediate, or resistant for each antibiotic tested following the CLSI guidelines and by measuring the zone of inhibition around the antibiotic disc using a ruler [27].

### 2.7. Statistical Analysis

Data collected in the field were recorded and stored in an Excel spreadsheet Version 2016. These data were then imported into SPSS Version 20 statistical software for analysis. Descriptive statistics were used to determine the frequency of virulent genes and the results of antimicrobial sensitivity.

## 3. Results

### 3.1. Bacteriological Result of *Staphylococcus aureus*

*S. aureus* was isolated from 64 (45.7%) milk samples. The proportion of isolation of *S. aureus* from milk of clinically and subclinically affected cows was 9 (34.6%) and 55 (48.2%), respectively (Table 2).

### 3.2. Occurrence of Virulence Genes

All 64 isolates were found positive for the nuc gene. Significant virulent genes were detected in enterotoxins (*seb* = 13 [20.3%], *sec* = 11 [17.2%], *seh* = 9 [14.1%], and *see* = 6 [9.4%]), *pvl* (pvl = 11 [17.2%]), *tst* (*tst* = 7 [10.9%]), and *hlb* (*hlb* = 7 [10.9%]) (Supporting file 1).

### 3.3. Antimicrobial Susceptibility Test Results

**Figure 2:**
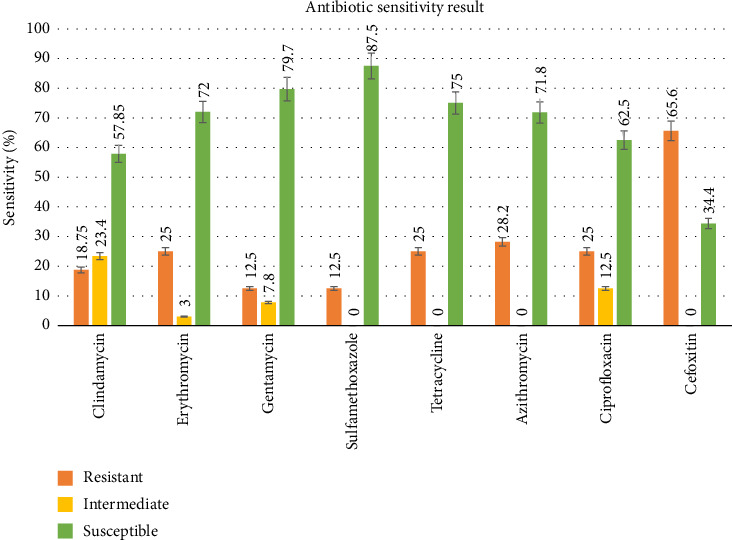
Antibiotic susceptibility profile of *S. aureus* isolates from bovine mastitis.

The isolates were highly susceptible to sulfamethoxazole (87.5%) and gentamycin (79.7%), followed by tetracycline (75%), erythromycin (72%), and azithromycin (71.8%). However, they were highly resistant to cefoxitin (65.6%), followed by erythromycin, tetracycline, and ciprofloxacin (25%) (Figure 2).

Multidrug resistance was observed in 23 isolates, showing resistance to at least one agent in three or more antimicrobial categories. The resistant isolates were recovered from the ranch and smallholder dairy farms, as indicated in Table 3.

## 4. Discussion

The frequency of isolation of *S. aureus* from the milk in the study area was 45.7%. An isolation rate of 45.7% indicates that nearly half of the milk samples tested were positive for *S*. *aureus*. A high isolation rate suggests that mastitis caused by *S*. *aureus* is a common issue in the study area, potentially affecting the overall health and productivity of the dairy herd. Increased prevalence of *S*. *aureus* in the region may be associated with inadequate cleaning of milking equipment and udders, incorrect milking techniques, overcrowded conditions, limited access to veterinary services and treatments, and poor farm management practices. With such a high prevalence, there may be increased use of antibiotics to treat mastitis, which can contribute to the development of antibiotic-resistant strains of *S*. *aureus*. The current finding was in line with the findings of Demissie, Menghistu, and Mitiku [28], who reported 42.31% in and around Wukro (Tigray). The finding was comparably higher than the study of Asmare and Kassa [29], Regasa et al. [30], Dereje et al. [31], *and* Abunna, Mengistu, and Abraha [32], who reported 39% in Sodo town, 15.3% in Mukaturi and Sululta town (Oromia), 30.9% in Holeta, and 21.13% in Addis Ababa City (Ethiopia), respectively. However, the present study was lower than Zenebe, Habtamu, and Endale [33], who reported 51.7% in Adigrat, Northern Ethiopia.

The isolation rate of *S. aureus* from clinical mastitis was 34.6%. The result was in line with the report by Kemal et al. [34], who reported 34.62% in and around Asella town. However, these results were higher than those reported by Gangwal et al. [35], who presented 24%. The 48.2% isolation rate of *S. aureus* from subclinical mastitis was in agreement with the report by Zenebe, Habtamu, and Endale [33] in Adigrat, who reported 49.43%. In contrast to the present finding, the higher prevalence was reported by Gianneechini et al. [36] and Abebe et al. [37], who reported 62.8% in Uruguay and 54.5% in Hawassa, respectively. However, the lowest prevalence of *S. aureus* isolated from subclinical mastitis was reported by Saeed et al. [38] at 16.5% in East Coast Malaysia and Workineh et al. [39] at 39.3% in Addis Ababa.

*S. aureus* species are adapted to survive in the udder and become sources of infection for healthy cows during milking. In turn, it will play a role in the variation of the prevalence. Transmission among cows increases when there is the absence of dry cow therapy, postmilking teat dipping, and the low culling rate of chronically infected cows [40].

The study detected virulent genes such as enterotoxins (*seb*, *sec*, *see*, and *seh*), *pvl*, *tsts*, and *hlb* that are associated with *S. aureus* pathogenicity. In this study, the most frequently identified virulence genes included enterotoxins (seb found in 13 [20.3%], *sec* in 11 [17.2%], *seh* in 9 [14.1%], and *see* in 6 [9.4%] samples), *pvl* (pvl detected in 11 [17.2%] samples), *tst* (tst present in 7 [10.9%] samples), and *hlb* (*hlb* also found in 7 [10.9%] samples). Supporting the present finding, tst genes were detected by Dorjgochoo et al. in a research conducted in Ulaanbaatar city, Mongolia [41]. Tegegne et al. conducted a study on bovine mastitis and found only the *clfA* and eta genes among the tested tsst-1, *hlb*, *eta*, *sea*, *clfA*, and *icaD* genes [42], which disagree with the current study. Contrary to our finding, a higher level of hemolysin gene was recorded in the research conducted by Wilson, Zishiri, and El Zowalaty [43]. The variation in the detection of virulence genes in *S. aureus* across different studies can be attributed to several factors. *S. aureus* can acquire virulence genes through mechanisms such as phage transduction, conjugation, and transformation. This leads to genetic diversity among strains. Different geographical regions may have distinct *S. aureus* strains with varying virulence gene profiles due to local environmental pressures and farming practices. Differences in the sensitivity and specificity of detection methods can also lead to variations in reported virulence gene frequencies.

All 64 confirmed isolates of *S. aureus* from mastitic milk were tested for antimicrobial susceptibility to eight selected antibiotics. The finding discovered 23 isolates resistant to three or more antibiotic groups. Higher MAR indexes were recorded in these isolates, indicating a greater risk of antibiotic resistance. The result of this study showed that *S. aureus* isolates were highly susceptible to certain tested antibiotics such as sulfamethoxazole (87.5%), gentamycin (79.7%), tetracycline (75%), erythromycin (72%), and azithromycin (71.8%). These susceptibility profiles were in line with studies by Choudhury et al. [44] in Assam (Northeast India), Nwankwo and Nasiru [45] in Kano (Northwest Nigeria), and Gentilini et al. [46] in Argentina. The high susceptibility of certain antibiotics may be due to infrequent use in the study area for treating *S. aureus* infections and as growth promoters for animal production. Relatively high resistant isolates were recorded for cefoxitin (65.6%), which is consistent with the studies of Choudhury et al. [44] in Assam (Northeast India) and Aetrugh et al. [47] in Libya. The resistance of *Staphylococcus aureus* to certain antibiotic groups in a specific region might be due to their frequent, long-term use of antibiotics and the distribution of the antibiotic-resistant gene among pathogens all over the world [48, 49]. The 65.6% resistance of isolates to cefoxitin indicates that the isolates recovered in our study are likely methicillin-resistant *Staphylococcus aureus* (MRSA). A limitation of this study is the lack of molecular characterization, such as phylogenetic analysis and MRSA detection. Thus, including phylogenetic analysis in future research will provide deeper insights into bacterial evolution, diversity, and epidemiology. Therefore, future studies should incorporate such analyses.

## 5. Conclusion

*Staphylococcus aureus* was identified as a common cause of mastitis. The isolates were confirmed to carry virulence genes by molecular methods. These isolates showed resistance to cefoxitin but were susceptible to sulfamethoxazole, gentamycin, tetracycline, erythromycin, and azithromycin. Multidrug-resistant strains were also detected in the study area.

## Figures and Tables

**Table 1:** Target genes and their primer sequences used for PCR detection of *Staphylococcus aureus*.

Gene	Primer sequence	Amplicon size (bp)	Annealing temperature (°C)	References
nuc	F: GCGATTGATGGTGATACGGTT	279	55	[16]
	R: AGCCAAGCCTTGACGAACTAAAGC			

sea	F: ATTAACCGAAGGTTCTGTAGA	552	54	[17]
	R: TTGCGTAAATCTGAA TT			

seb	F: TGTATGTATGGAGGTGTAAC	270	50	[18]
	R: ATAGTGACGAGTTAGGTA			

sec	F: CCACTTTGATAATGGGAACTTAC	270	56	[19]
	R: GATTGGTCAAACTTATCGCCTGG			

sed	F: CTAGTTTGGTAATATCTCCT	317	55	[20]
	R: TAATGCTATATCTTATAGGG			

see	F: TAGATAAAGTTAAAACAAGC	170	55	[21]
	R: TAACTTACCGTGGACCCTTC			

seh	F: CACATCATATGCGAAAGCAGA	617	55	[22]
	R: CCTTTTAAATCATAAATGTCGAATGA			

pvl	F: TTACACAGTTAAATATGAAGTGAACTGGA	118	60	[23]
	R: AGCAAAAGCAATGCAATTGATG			

tst	F: ACCCCTGTTCCCTTATCATC	326	57	[24]
	R: TTTTCAGTATTTGTAACGCC			

hlb	F: GTGCACTTACTGACAATAGTGC	309	55	[25]
	R: GTTGATGAGTAGCTACCTTCA			

**Table 2:** Frequency of isolation of *S. aureus* among clinical and subclinical mastitis milk samples.

Type of mastitis	No. of sample	Farming system *N* (%)		*Staphylococcus aureus* isolates *N* (%)		*Non-Staphylococcus aureus* isolates *N* (%)	
		Ranch	Small	Ranch	Small	Ranch	Small
Clinical mastitis	26	17 (65.4)	9 (34.6)	6 (23.1)	3 (11.5)	12 (46.2)	5 (19.2)
Subclinical mastitis	114	69 (60.5)	45 (39.5)	36 (31.6)	19 (16.7)	38 (33.3)	21 (18.4)

*Note: N*, number; Ranch, Chagni ranch; Small, smallholder dairy farms.

**Table 3:** Multidrug-resistant isolates and their multiple antibiotic resistance index (MARI).

Source	Isolate id	No. of antibiotics to which the isolate was resistant (*a*)	MAR index (*a/b*)
Ranch	04R	5 (CLI–ERY–TET–AZI–CEF)	0.63
	07R	6 (CLI–ERY–GEN–SMX–AZT–CEF)	0.75
	16R	4 (GEN–SMX–TET–AZI)	0.5
	17R	5 (CLI–GEN–AZI–CIP–CEF)	0.63
	28R	3 (CLI–ERY–CEF)	0.38
	37R	5 (ERY–TET–AZI–CIP–CEF)	0.63
	52R	4 (TET–AZI–CIP–CEF)	0.5
	102R	4 (TET-–AZI–CIP–CEF)	0.5
	106R	6 (CLI–ERY–TET–AZI-–CIP–CEF)	0.75
	113R	5 (GEN–SMX-–TET–CIP–CEF)	0.63
	119R	6 (CLI–ERY–SMX–TET–AZI–CEF)	0.75
	120R	5 (ERY–TET-–AZI–CIP–CEF)	0.63
	122R	6 (GEN–SMX–TET–AZI–CIP–CEF)	0.75
	130R	6 (CLI–ERY–SMX–AZI–CIP–CEF)	0.75
	138R	4 (TET–AZI-–CIP–CEF)	0.5

Smallholder	13S	5 (SMX–TET–AZI–CIP–CEF)	0.63
	38S	6 (CLI–GEN–SMX–TET–AZI–CIP)	0.75
	66S	6 (CLI–GE–TET–AZI–CIP–CEF)	0.75
	91S	4 (CLI–ERY–TET–CEF)	0.5
	104S	5 (CLI-ERY–AZI–CIP–CEF)	0.63
	112S	3 (AZI–CIP–CEF)	0.38
	124S	4 (CLI–GEN–TET–CEF)	0.5
	131S	3 (ERY–CIP–CEF)	0.38

*Note: b*, the number of antibiotics to which the isolate was tested (*n* = 8). AZI, azithromycin; CEF, cefoxitin; CIP, ciprofloxacin; CLI, clindamycin; ERY, erythromycin; GEN, gentamycin; SMX, sulfamethoxazole; TET, tetracycline.

Abbreviations: *R*, ranch; *S*, small.

## Data Availability

The data that support the findings of this study are available from the corresponding author upon reasonable request.

## Supporting Information

The Supporting file includes gel electrophoresis images that display the virulent genes of *S. aureus*.
