# Supplementary material for: Detection of Virulence Genes and Antimicrobial Susceptibility Profiles of Staphylococcus aureus Isolates From Bovine Mastitis in Chagni, Northwestern Ethiopia
Source: Vet Med Int. 2025 Feb 17;2025:6473601. doi: 10.1155/vmi/6473601 (PMC11850071; doi:10.1155/vmi/6473601)
Supplement: Supporting Information — Additional supporting information can be found online in the Supporting Information section. [file 6473601.f1.docx]

Supplementary file 1: Gel pictures of the virulent genes of *S. aureus*


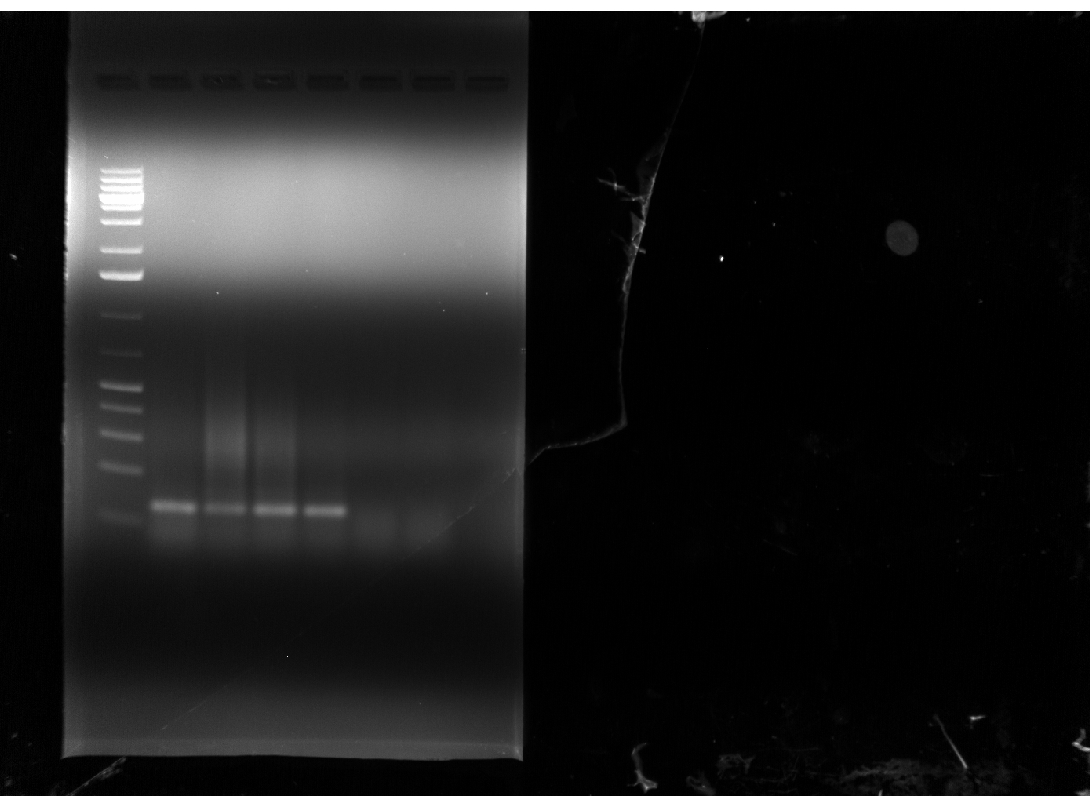


PVL-118 bp


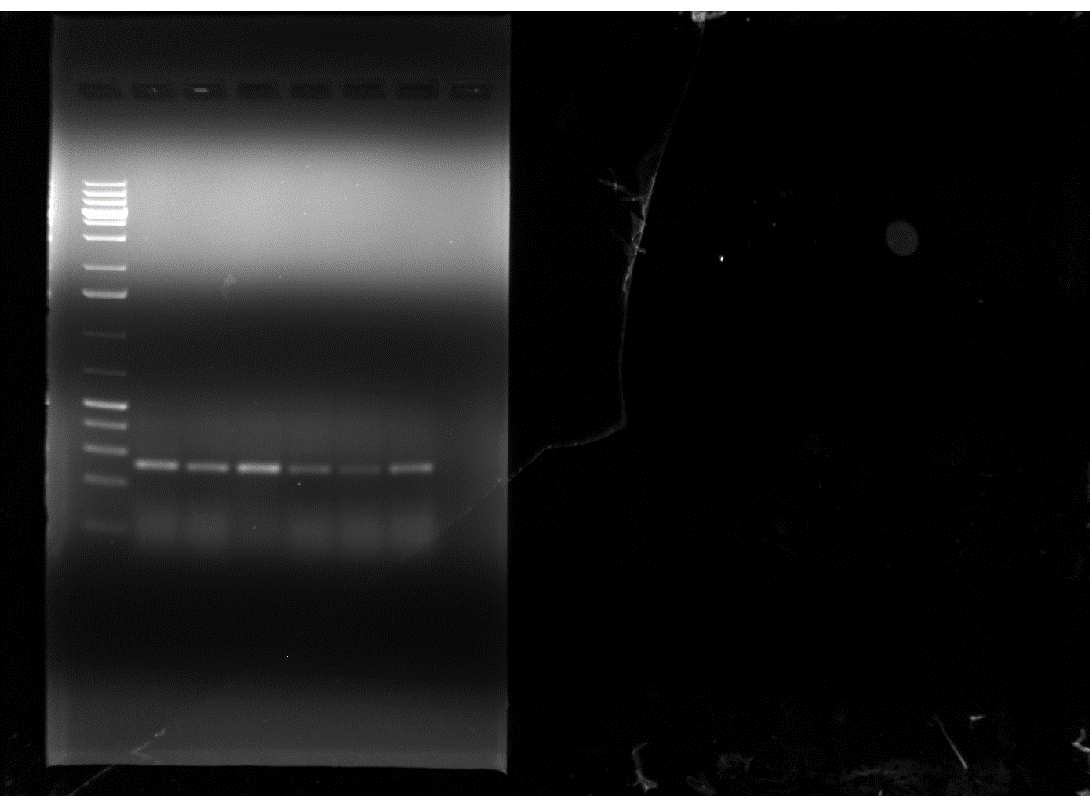


Seb-270bp


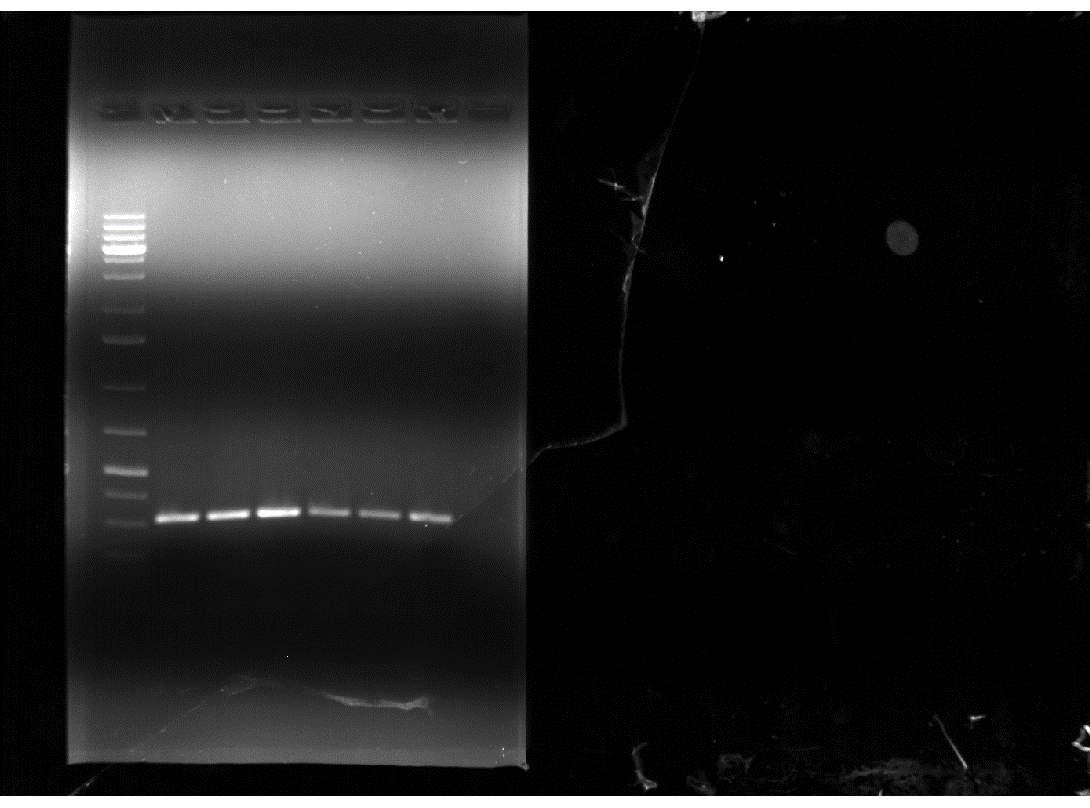


Hlb-309bp


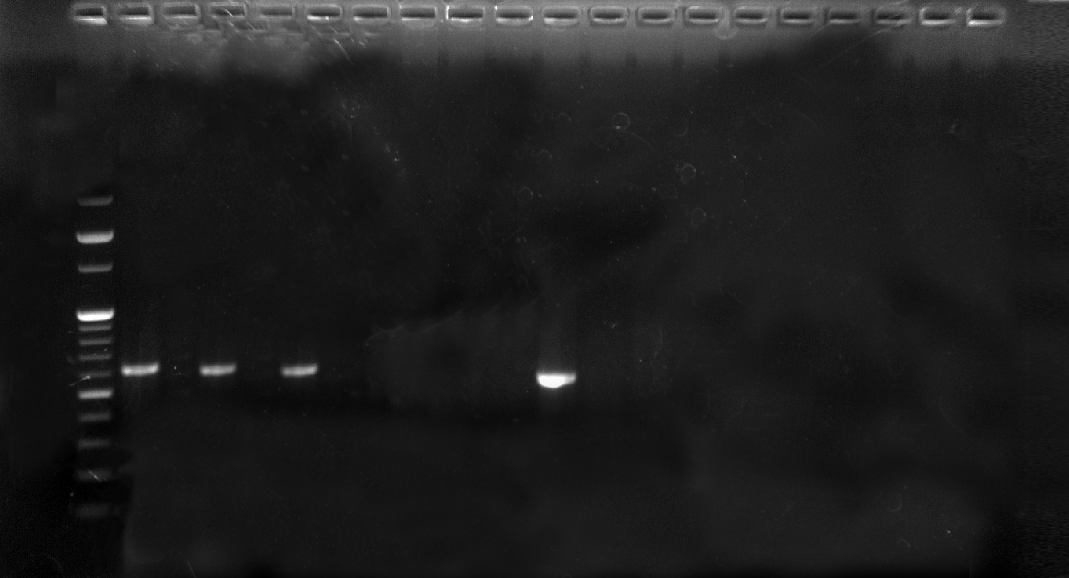


Seh-617bp


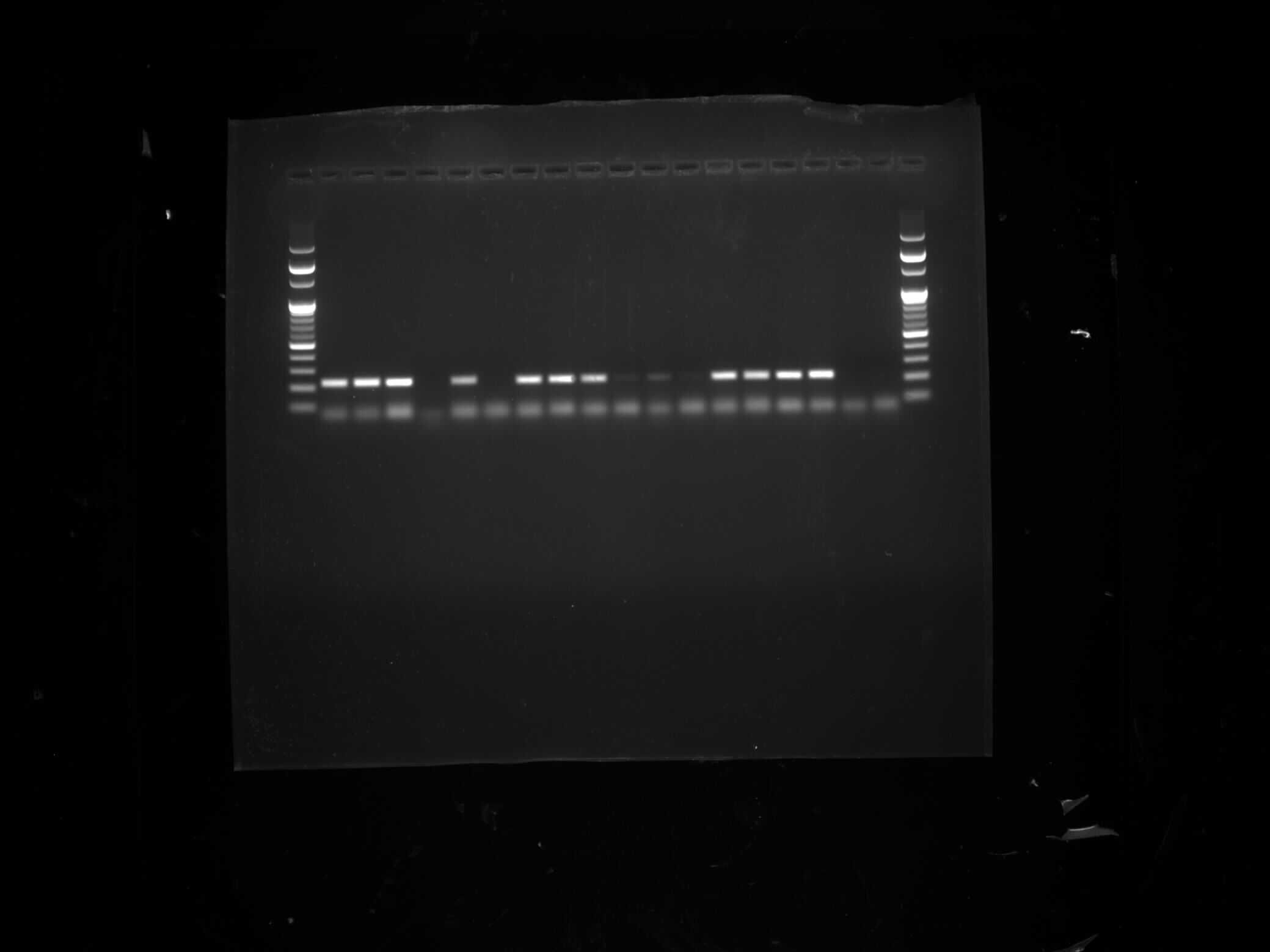


Sec-270bp


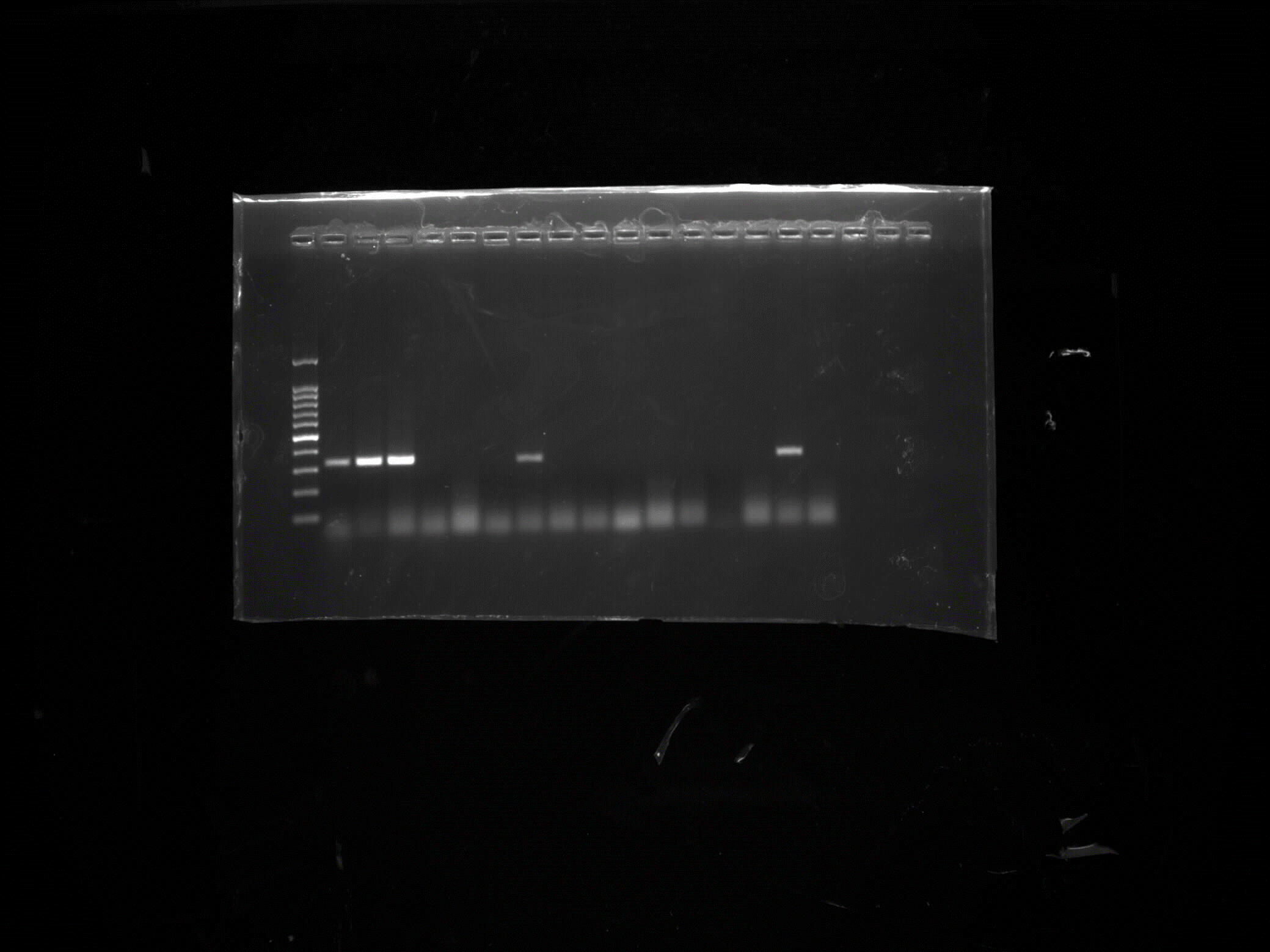


Tst-326bp


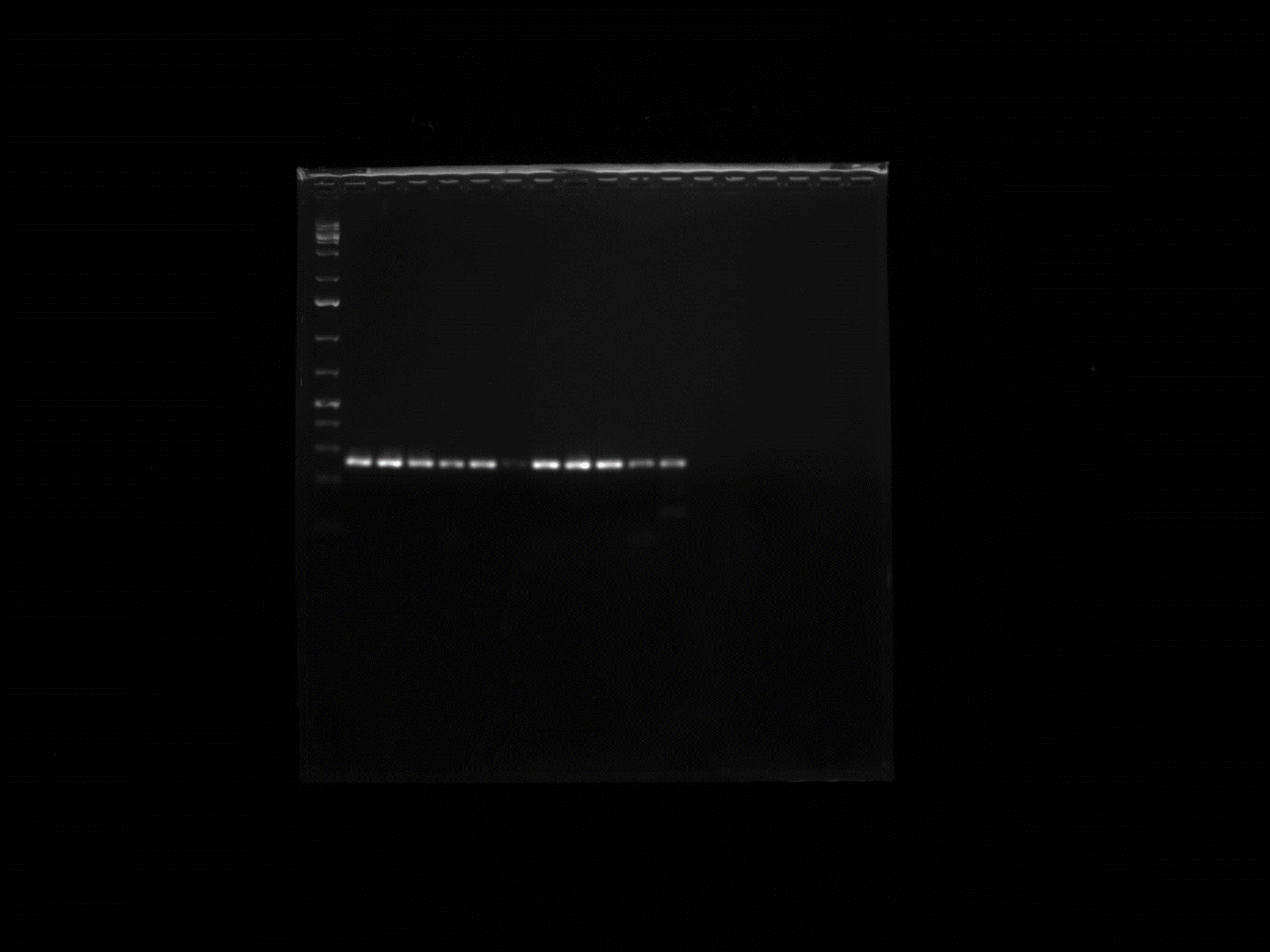


Nuc-279bp


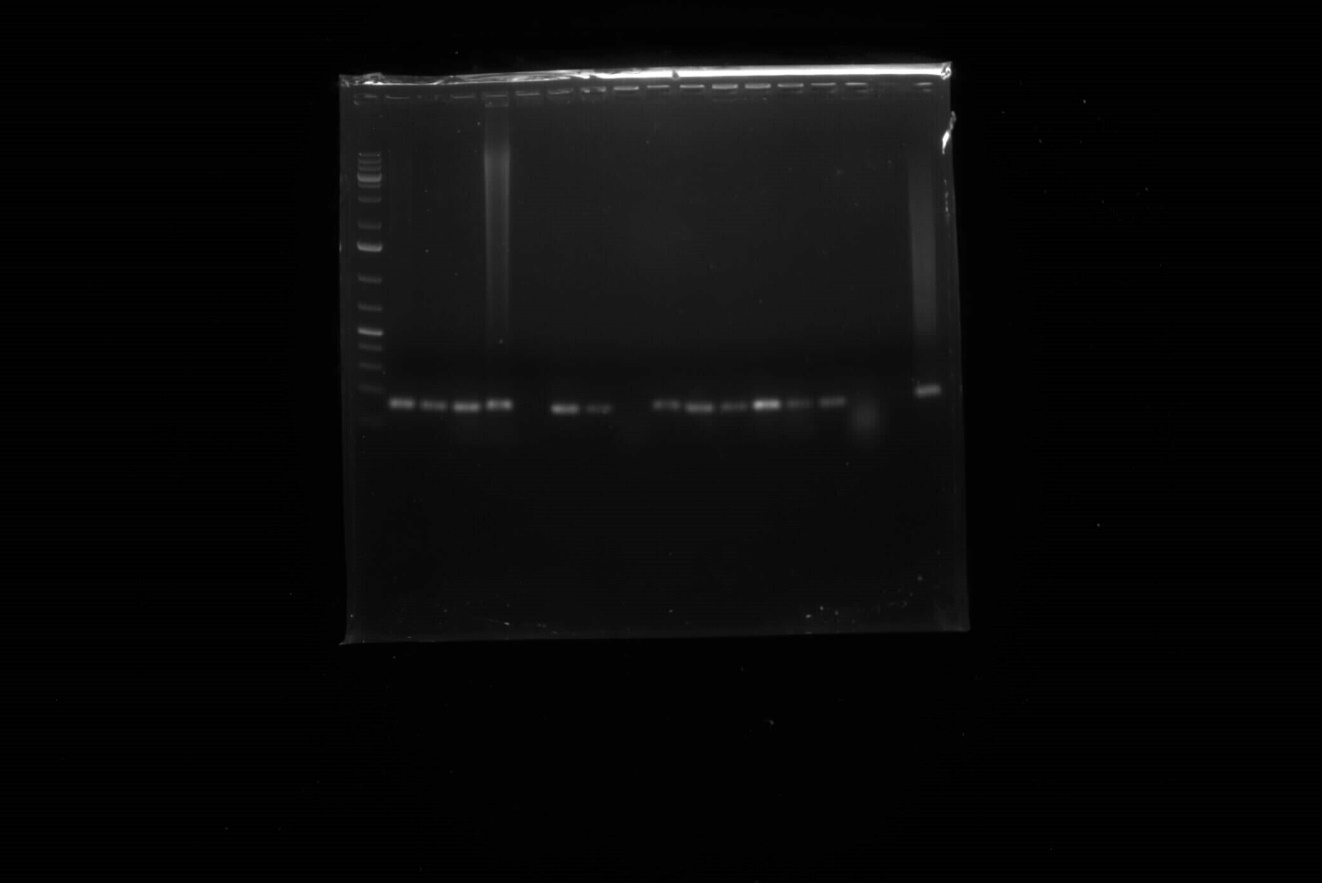


See-170bp
